# Supplementary material for: Acidity-activatable upconversion afterglow luminescence cocktail nanoparticles for ultrasensitive in vivo imaging
Source: Nat Commun. 2024 Mar 8;15:2124. doi: 10.1038/s41467-024-46436-z (PMC10923940; doi:10.1038/s41467-024-46436-z)
Supplement: Supplementary file 1 — Supplementary Information [file 41467_2024_46436_MOESM1_ESM.pdf]

Supplementary Information

Acidity-activatable upconversion afterglow luminescence cocktail nanoparticles for ultrasensitive in vivo imaging

Yue Jiang[1,3], Min Zhao[1,3], Jia Miao[1], Wan Chen[1], Yuan Zhang[1], Minqian Miao[1], Li Yang[1], Qing Li[1] & Qingqing Miao[1, 2*]

[1]State Key Laboratory of Radiation Medicine and Protection, School for Radiological and Interdisciplinary Sciences (RAD-X), Collaborative Innovation Center of Radiation Medicine of Jiangsu Higher Education Institutions, Soochow University, Suzhou 215123, China

[2]School of Nuclear Science and Technology, University of Science and Technology of China, Hefei 230026, China

*e-mail: qqmiao@suda.edu.cn

[3]These authors contributed equally: Yue Jiang, Min Zhao.

Table of contents

1. Supplementary Methods.. 2

2. Supplementary Figures and Tables 3

3. Supplementary References.. 18

1. Supplementary Methods

Materials

All chemicals and solvents were purchased from commercial suppliers in analytical grade and used without further purification unless otherwise stated. C_{18}-PEG_{2000}-NH_2 was purchased from Shenzhen MeloPEG Technology Co. LTD. 2,3-dimethylmaleic anhydride (DA) and succinic anhydride (SA) were purchased from Aladdin. Poly[2-methoxy-5-(2'-ethylhexyloxy)-1,4-phenylene vinylene] (MEHPPV) (Mn (MEHPPV) = 40,000~70,000 g/mol) and poly(styrene)-block-poly(acrylic acid) (PS-*b*-PAA) (Mn (PS) = 2700~3300 g/mol, Mn (PAA) = 4000~6000 g/mol, PDI ≤ 1.1) were purchased from Sigma-Aldrich. Silicon 2,3-naphthalocyanine bis(trihexylsilyloxide) (NCBS) was purchased from Alfa Chemistry. Rose bengal (RB) was purchased from Sigma-Aldrich (Shanghai) Trading Co., Ltd. 2-Ethylhexyl bromide was purchased from Shanghai Aladdin Biochemical Technology Co., Ltd. All cell lines (4T1, 4T1-luc, HepG2, and B16F10) were purchased from Stem Cell Bank, Chinese Academy of Sciences (Shanghai, China). RPMI 1640 medium and DMEM medium were purchased from HyClone. Penicillin-streptomycin and fetal bovine serum (FBS) were purchased from Gibco. Cell Counting Kit-8 (CCK-8) was purchased from APExBIO. Water was supplied by Milli-Q Plus System (Millipore Corporation, Breford, USA). We confirm that all unique materials used are readily available from the authors or from standard commercial sources.

Materials characterization

Proton nuclear magnetic resonance (^{1}H NMR) spectra were measured by a Bruker Avance II 400 MHz NMR and Fourier transform infrared spectroscopy (FTIR) were measured by a Bruker INVENIO-R. Dynamic light scattering (DLS) was recorded on a Zetasizer Nano ZS (Nano ZS90, Malvern). TEM images were captured using an FEI Tecnai F20 transmission electron microscope operating at an acceleration voltage of 80 kV. UV-Vis and fluorescence spectra were recorded on a PerkinElmer Lambda 35 and an Edinburgh FLS980 spectrofluorometer, respectively. Fluorescence, afterglow luminescence images and afterglow luminescence spectra were recorded on an IVIS Spectrum imaging system (PerkinElmer, Inc.). A Fiber Optic Illuminator equipped with a 150 W halogen lamp was used as the light source for generating the afterglow luminescence. A fiber coupled with an 808 nm laser system was purchased from Changchun New Industries Optoelectronics Tech. Co. Ltd.

FTIR measurements

The freeze-dried C_{18}-PEG_{2000}-DA and C_{18}-PEG_{2000}-SA were thoroughly mixed with potassium bromide powder and ground into powder in a mortar. Then the powder mixture is pressed into transparent discs by a tablet press to allow infrared light to penetrate. Finally, the discs are placed in a Fourier transform infrared absorption spectrometer for analysis. Note that potassium bromide discs need to be measured to get a background signal before the measurement of samples.

Preparation of ASN, C-ASN, Bio-ASN, AIN, Bio-AIN, and R-AIN

A mixed THF solution (1 mL) containing MEHPPV (0.25 mg/mL) and C_{18}-PEG_{2000}-DA (2 mg/mL) was used to prepare ASN by rapidly injecting it into 1 × HEPES (9 mL, pH = 8.5) under continuous vigorous sonication. C-ASN were prepared in the same way as ASN. Bio-ASN nanoparticles were obtained by mixing C_{18}-PEG-Biotin (0.25 mg/mL), MEHPPV (0.25 mg/mL), and C_{18}-PEG_{2000}-DA (2 mg/mL) in THF solution (1 mL), followed by the same procedures as above. For AIN, NCBS (0.05 mg/mL) and PS-*b*-PAA (0.4 mg/mL) were mixed in THF solution (1 mL) followed by the same sonication step. After sonication, THF was slowly removed with by a gentle nitrogen flow. The aqueous solution was filtered through a polyethersulfone (PES) syringe-driven filter (0.22 μm) (Millipore), and washed three times using 30 K centrifugal filter units (Millipore) under centrifugation at 1,300 g for 15 min. Bio-AIN was prepared by mixing C_{18}-PEG-Biotin (0.05 mg/mL), NCBS (0.05 mg/mL), and PS-*b*-PAA (0.4 mg/mL) in THF solution (1 mL), followed by sonication and other same steps as above. R-AIN nanoparticles were obtained by mixing alkylated-rose bengal (a-RB, 0.01 mg/mL, Supplementary Fig. 15, 35), NCBS (0.05 mg/mL), and PS-*b*-PAA (0.4 mg/mL) in THF solution (1 mL), followed by the same procedures as above. The concentrations of nanoparticles were determined by UV-Vis absorption according to their absorption coefficients. The ASN and C-ASN solutions were finally concentrated to 0.5 mg/mL (based on the mass of MEHPPV) and AIN and R-AIN solutions were concentrated to 0.25 mg/mL (based on the mass of NCBS) by ultrafiltration and stored in dark at ~ 4 °C.

Afterglow and fluorescence luminescence imaging

Afterglow and fluorescence luminescence imaging were performed on an IVIS Spectrum imaging system in bioluminescence and fluorescence modes, respectively. For acquisition of afterglow luminescence images, samples were pre-illuminated by 808 nm laser at a power density of 1 W/cm^2 or white light at a power density of 0.1 W/cm^2 for 1 min unless otherwise noted. In vitro afterglow signals were collected for 10 s with an open filter. In vitro fluorescence signals were collected for 5 s with an excitation wavelength at 480 nm and emission wavelength at 580 nm. The afterglow spectra were collected for 5 s with specific emission filters. For in vivo experiments, the afterglow images were recorded for 30 s with an open filter, the fluorescence images were recorded for 5 s with an excitation wavelength at 480 nm and

emission wavelength at 580 nm. The afterglow and fluorescence intensities were quantified by measuring the signal intensity of the region of interest (ROI) using Living Imaging 4.5 Software.

Cell culture

4T1 cancer cells were cultured in RPMI medium 1640 (BasalMedia) supplemented with 10% (v/v) fetal bovine serum (FBS, HyClone) and 1% (v/v) penicillin/streptomycin antibiotics. HepG2 and B16F10 cancer cells were cultured in DMEM (BasalMedia) supplemented with 10% (v/v) FBS and 1% (v/v) penicillin/streptomycin antibiotics. The cells were placed in an incubator with 5% CO_2 and 95% humidified air at 37 °C.

Subcellular localization study

HepG2 cells were seeded on a confocal dish (35 mm) with 1×10^4 cells per dish with 1 mL medium overnight. HepG2 cells were pretreated with ASN (40 μg/mL) or R-AIN (40 μg/mL) for 12 h, respectively. Next, the medium was removed and the cells were washed with PBS for three times. Then, the cells were then stained with commercial Lyso Green Tracker (10 μM), Mito Green Tracker (10 μM) and Hoechst 33342 (10 μM) for the nuclei. Fluorescence images were obtained on CLSM. For Lyso Green Tracker and Mito Green Tracker, an excitation wavelength was 488 nm with emission wavelengths at 510 nm ± 10 nm. For ASN, an excitation wavelength was 488 nm with emission wavelengths at 580 nm ± 10 nm. For R-AIN, an excitation wavelength was 559 nm with emission wavelengths at 580 nm ± 10 nm.

Cytotoxicity assay

HepG2 cancer cells were seeded in 96-well plates (8000 cells in 100 μL supplemented medium per well) and incubated for 24 h. Aqueous dispersions of ASN or C-ASN (final concentrations 1, 2, 5, 10, 20, and 30 μg/mL) and AIN (final concentrations 0.1, 0.2, 0.5, 1.0, 2.0, and 3.0 μg/mL) were introduced into the culture medium and incubated for 24 h. The cell culture medium was removed, and the fresh medium (100 μL per well) mixed with CCK-8 (5 mg/mL, 10 μL per well) was then added in the wells. After incubation for 4 h, the absorbance of CCK-8 at 450 nm was recorded by EnSpire multimode plate reader (PerkinElmer). Cell viability was calculated according to the ratio of absorbance of experimental well to that of the control cell well.

Blood circulation

ASN (8 mg/kg) and AIN (0.8 mg/kg) were i.v. injected into mice, respectively. At different time points post-injection, blood samples were collected from the retinal vein for fluorescence assays. The blood samples were diluted by four-fold with 1 × HEPES buffer. The contents of ASN and AIN in the blood samples were determined through fluorescence by IVIS Spectrum imaging system (PerkinElmer, Inc.) with an excitation at 480 nm and emission at 580 nm (ASN), and an excitation at 720 nm and emission at 790 nm (AIN), respectively.

Biodistribution method

The mice were euthanized by CO_2 asphyxiation 48 h after administration of ALCNs and C-ALCNs (n = 3). Major organs were collected and then placed onto black paper. All organs were pre-irradiated with an 808 nm laser (1 W/cm^2) or white light (0.1 W/cm^2) for 1 min, and the afterglow luminescence images were acquired for 10 s with an open filter using IVIS Spectrum imaging system. The fluorescence images were acquired for 5 s under an excitation wavelength at 480 nm and emission wavelength at 580 nm. The afterglow and fluorescence luminescence intensities for each individual organ were analyzed by the ROI analysis using the Living Image 4.5 Software.

Histology

After being fixed with 4% paraformaldehyde (PFA), the organs were dehydrated in ethanol solution and embedded in paraffin prior to 10 μm sectioning. Histology samples were stained by hematoxylin and eosin under standard protocols. Images were obtained on a confocal microscope (FV1200, Olympus).

2. Supplementary Figures and Tables

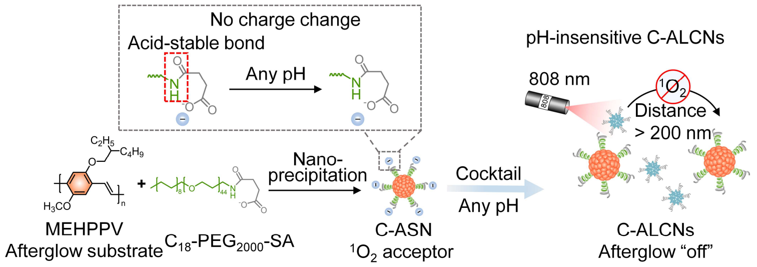

Supplementary Fig. 1. Schematic illustration for the pH-insensitive nanoprobe, C-ALCNs. Schematic illustration of the preparation of C-ASN through nanoprecipitation. No charge changes occurred at any pH and thus the $^1O_2$ transfer

from AIN to C-ASN was inhibited, leading to inactivated afterglow signal.

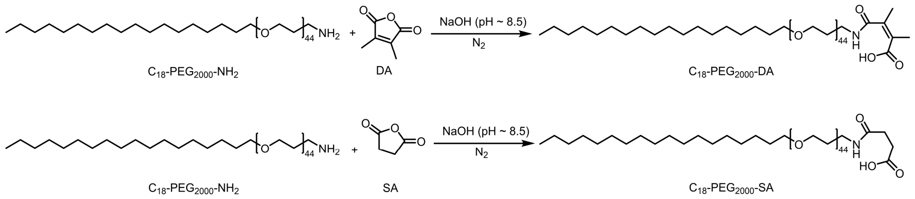

Supplementary Fig. 2. Synthetic routes of C_{18}-PEG$_{2000}$-DA and C_{18}-PEG$_{2000}$-SA.

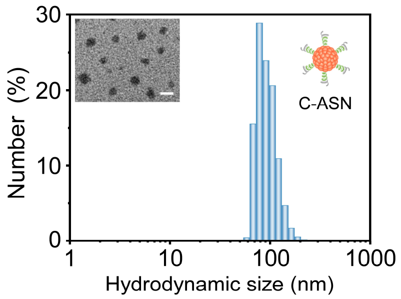

Supplementary Fig. 3. Characterizations of C-ASN. Average hydrodynamic diameters and representative TEM image of C-ASN in 1× HEPES buffer (pH = 7.4, the scale bar represents 100 nm). Source data are provided as a Source Data file.

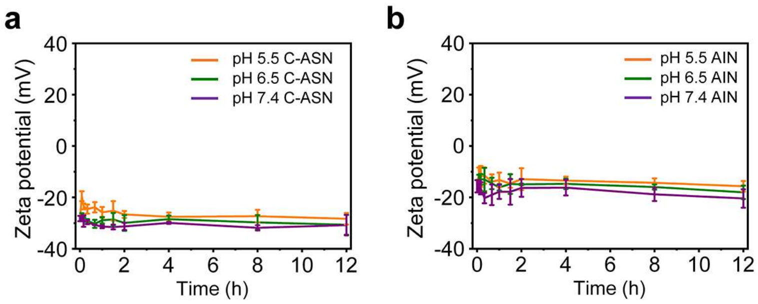

Supplementary Fig. 4. Study of the zeta potential of nanoparticles. a, b The time-dependence of surface zeta potential of C-ASN (10 μg/mL) (a) and AIN (2 μg/mL) (b) at different pH values in 1 × HEPES buffer. The zeta potential values of pH 5.5 and 6.5 at 0 h were based on that of pH 7.4. Data are presented as mean ± s.d. (n = 3 independent experiences). Source data are provided as a Source Data file.

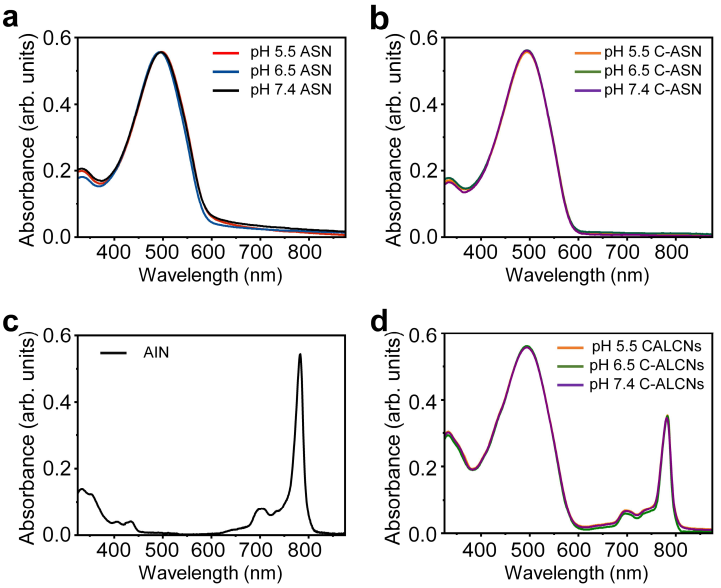

Supplementary Fig. 5. Comparison of absorption properties of nanoparticles. a-d UV-Vis absorption spectra of ASN (10 μg/mL) (a), C-ASN (10 μg/mL) (b), AIN (2 μg/mL) (c), and C-ALCNs (10 μg/mL C-ASN, and 1 μg/mL AIN) (d) at different pH values in 1 × HEPES buffer. Source data are provided as a Source Data file.

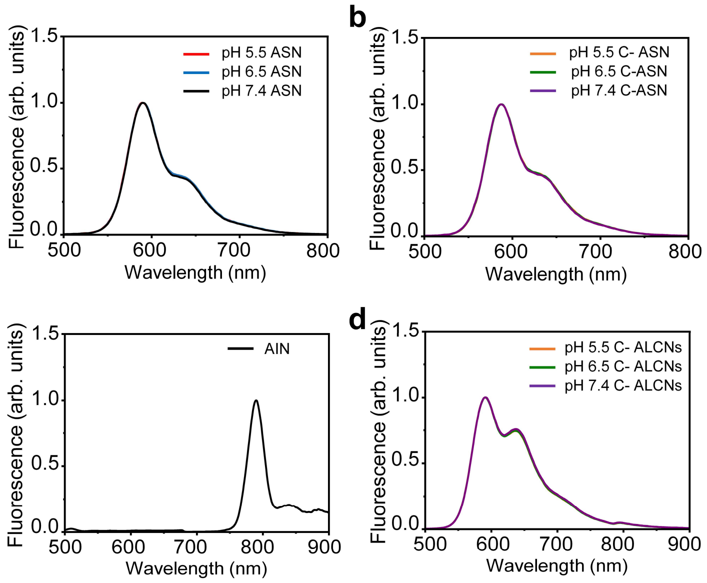

Supplementary Fig. 6. Comparison of fluorescence properties of nanoparticles. a, b Normalized fluorescence spectra of ASN (a) and C-ASN (b) under excitation at 480 nm at different pH values in 1 × HEPES buffer. **c** Normalized fluorescence spectra of AIN under excitation at 430 nm at different pH values in 1 × HEPES buffer. **d** Normalized fluorescence spectra of C-ALCNs under excitation at 480 nm at different pH values in 1 × HEPES buffer. Source data are provided as a Source Data file.

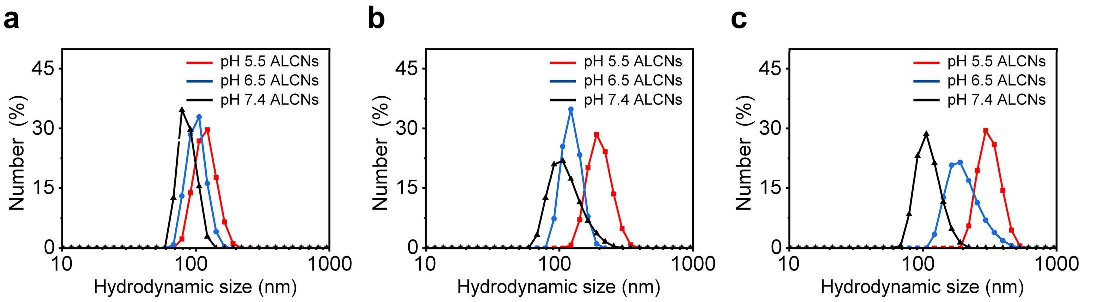

Supplementary Fig. 7. The size changes of ALCNs with different concentrations during pH-responsive process. a-c The hydrodynamic sizes of ALCNs (5 μg/mL ASN and 0.5 μg/mL AIN) (a), ALCNs (10 μg/mL ASN and 1 μg/mL AIN) (b), and ALCNs (20 μg/mL ASN and 2 μg/mL AIN) (c) at different pH values in 1 × HEPES buffer. Source data are provided as a Source Data file.

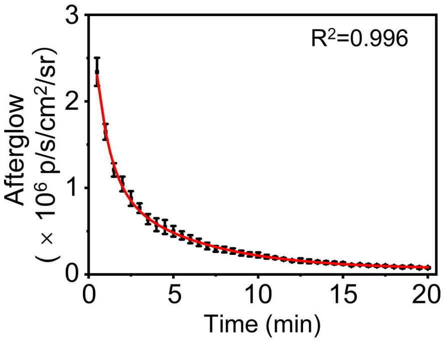

Supplementary Fig. 8. Afterglow luminescence decay of ALCNs. Afterglow luminescence decay of ALCNs (20 μg/mL ASN and 2 μg/mL AIN) recorded with 808 nm laser pre-irradiation (1 W/cm^2) in 1 × HEPES buffer. Data are presented as mean ± s.d. (n = 3 independent experiences). Source data are provided as a Source Data file.

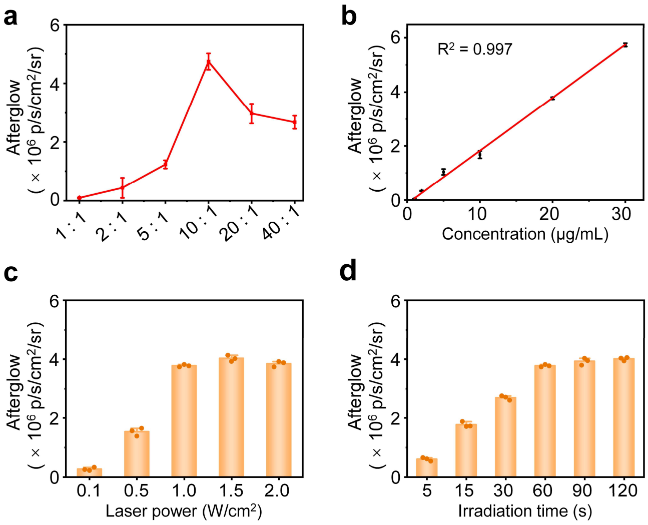

Supplementary Fig. 9. The afterglow intensity of ALCNs under different conditions. a The concentration ratio of ASN and AIN corresponds to the afterglow intensities under 808 nm laser pre-irradiation in 1 × HEPES buffer (pH 5.5). **b** Fitted calibration curve of the afterglow intensity of ALCNs after irradiation by 808 nm (1 W/cm^2) laser as a function of the concentration in 1 × HEPES buffer (pH 5.5). **c** Afterglow luminescence of ALCNs (20 μg/mL ASN and 2 μg/mL AIN) after illumination by 808 nm laser at different power densities in 1 × HEPES buffer (pH 5.5). **d** Afterglow luminescence of ALCNs (20 μg/mL ASN and 2 μg/mL AIN) at different light irradiation time by 808 nm laser (1 W/cm^2) in 1 ×

HEPES buffer (pH 5.5). Data are presented as mean ± s.d. (n = 3 independent experiences). Source data are provided as a Source Data file.

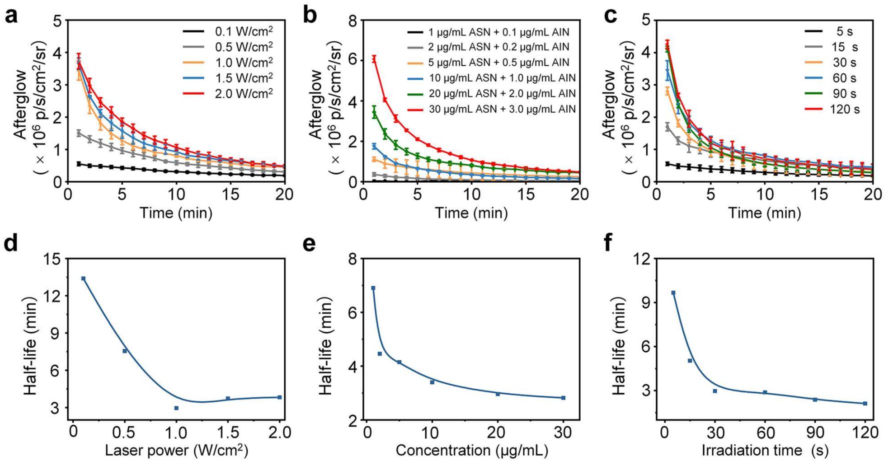

Supplementary Fig. 10. The afterglow duration of ALCNs under different conditions. a Afterglow luminescence attenuation curve of ALCNs (20 μg/mL ASN and 2 μg/mL AIN) after illumination by 808 nm laser at different power densities within 20 min in 1 × HEPES buffer (pH 5.5). **b** Afterglow luminescence attenuation curve of ALCNs after lamination by 808 nm (1 W/cm^2) laser as a function of the concentration within 20 min in 1 × HEPES buffer (pH 5.5). **c** Afterglow luminescence attenuation curve of ALCNs (20 μg/mL ASN and 2 μg/mL AIN) at different light irradiation time by 808 nm laser at power density of 1 W/cm^2 within 20 min in 1 × HEPES buffer (pH 5.5). **d-e** Corresponding half-life changes as a function of different conditions. Data are presented as mean ± s.d. (n = 3 independent experiences). Source data are provided as a Source Data file.

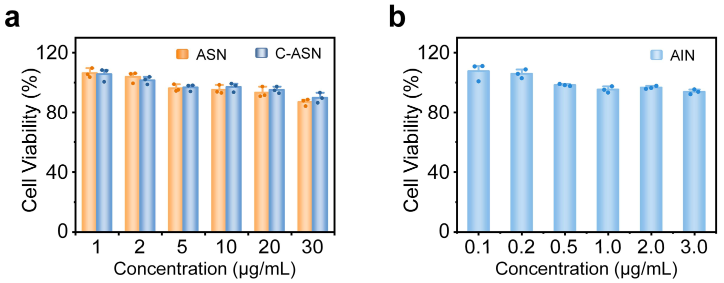

Supplementary Fig. 11. Cytotoxicity of nanoparticles. Cell viabilities of HepG2 cells treated with different concentrations of ASN and C-ASN (a), and AIN (b) for 24 h. Data are presented as mean ± s.d. (n = 3 biologically independent samples). Source data are provided as a Source Data file.

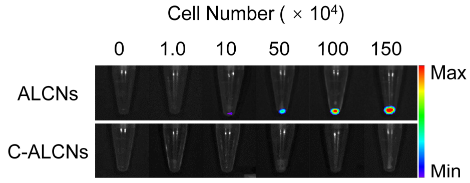

Supplementary Fig. 12. Afterglow imaging of HepG2 cells. Afterglow images under 808 nm laser pre-irradiation of ALCNs and C-ALCNs (30 μg/mL ASN or C-ASN, and 3 μg/mL AIN) incubated with different numbers of HepG2 cells in 1× HEPES buffer (pH = 5.5).

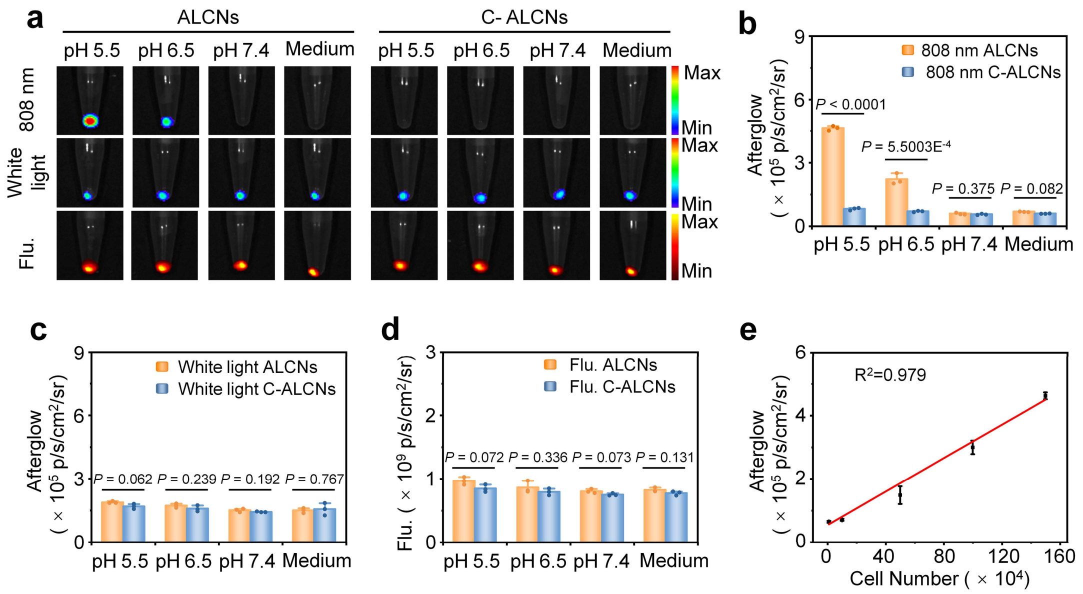

Supplementary Fig. 13. In vitro afterglow luminescence imaging capability of ALCNs towards 4T1 cells. a Afterglow luminescence images of cell pellets (about 4 × 10^6 4T1 cells per cell dish) after incubation with ALCNs or C-ALCNs (30 μg/mL ASN or C-ASN, and 3 μg/mL AIN) at different pH values and without pH adjustment under 808 nm pre-irradiation (upper panel) or white light pre-irradiation (middle panel), and corresponding fluorescence images (bottom panel). The fluorescence images were acquired with excitation at 480 nm and emission wavelength at 580 nm. **b-d** Quantification of afterglow intensities under 808 nm laser pre-irradiation (b), under white light pre-irradiation (c), fluorescence intensities (d) of 4T1 cells incubation of ALCNs and C-ALCNs. **e** Linear fitting curve of afterglow intensities with different cell numbers under 808 nm laser pre-irradiation of ALCNs in 1 × HEPES buffer (pH = 5.5). Data are presented as mean ± s.d. and analyzed by the Student's two-sided *t* test (*n* = 3 biologically independent samples). Source data are provided as a Source Data file.

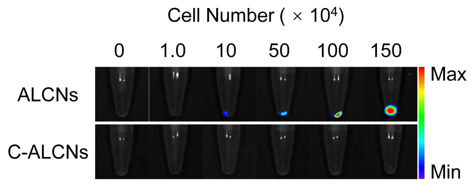

Supplementary Fig. 14. Afterglow imaging of 4T1 cells. Afterglow images under 808 nm laser pre-irradiation of ALCNs and C-ALCNs (30 μg/mL ASN or C-ASN, and 3 μg/mL AIN) incubated with different numbers of 4T1 cells in 1× HEPES buffer (pH = 5.5).

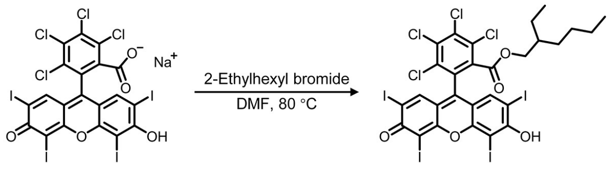

RB a-RB

Supplementary Fig. 15. Synthetic route of a-RB.

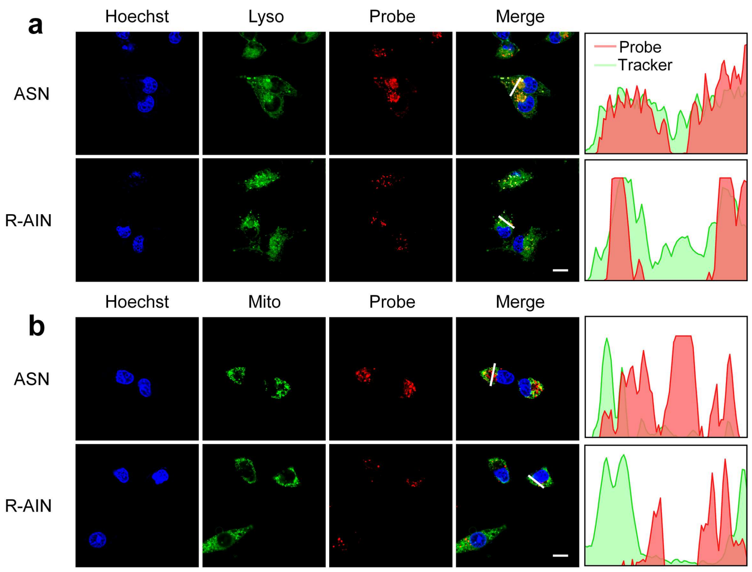

Supplementary Fig. 16. Subcellular localization study of ALCNs. Confocal fluorescence images of HepG2 cells treated with ASN (40 μg/mL), R-AIN (40 μg/mL) and Hoechst 33342 (10 μM), which were co-stained with commercial Lyso Green Tracker (10 μM) (a), Mito Green Tracker (10 μM) (b), respectively. For Lyso Green Tracker and Mito Green Tracker, an excitation wavelength was 488 nm with emission wavelengths at 510 nm ± 10 nm. For ASN, an excitation wavelength was 488 nm with emission wavelengths at 580 nm ± 10 nm. For R-AIN, an excitation wavelength was 559 nm with emission wavelengths at 580 nm ± 10 nm. Line-scan analyses of merged fluorescence images in (a) and (b) were performed by Image J. The scale bar represents 10 μm.

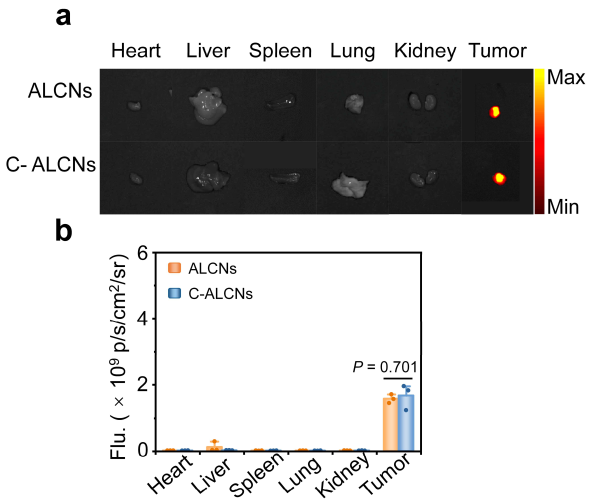

Supplementary Fig. 17. Study of biodistribution. a Ex vivo fluorescence images of various organs and tissues from an 4T1-xenograft subcutaneous tumor-bearing mouse intratumor injection of ALCNs and C-ALCNs (1 mg/kg ASN or C-ASN, and 0.1 mg/kg AIN) at 48 h with an excitation at 480 nm and emission at 580 nm. **b** Quantification of fluorescence intensities of organs and tissues. Data are presented as mean ± s.d. and analyzed by one-way ANOVA (n = 3 mice each group). Source data are provided as a Source Data file.

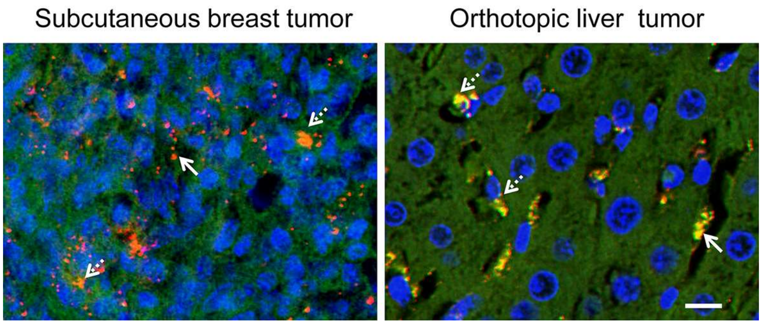

Supplementary Fig. 18. Fluorescence imaging of tumor sections. Fluorescent images of 4T1 subcutaneous tumor and HepG2 orthotopic tumor sections after treatment with ALCNs by Digital pathology scanner. ASN (green, 1 mg/kg for subcutaneous tumor, and 4 mg/kg for orthotopic tumor, with an excitation at 490 nm and emission at 570 ± 10 nm), AIN (red, 0.1 mg/kg for subcutaneous tumor, and 0.4 mg/kg for orthotopic tumor, with an excitation at 750 nm and emission at 780 ± 10 nm), nucleus (blue, 10 μM, with an excitation at 350 nm and emission at 420 ± 10 nm). The scale bar represents 5 μm. Dashed and solid arrows indicate the intracellular and extracellular co-localization, respectively.

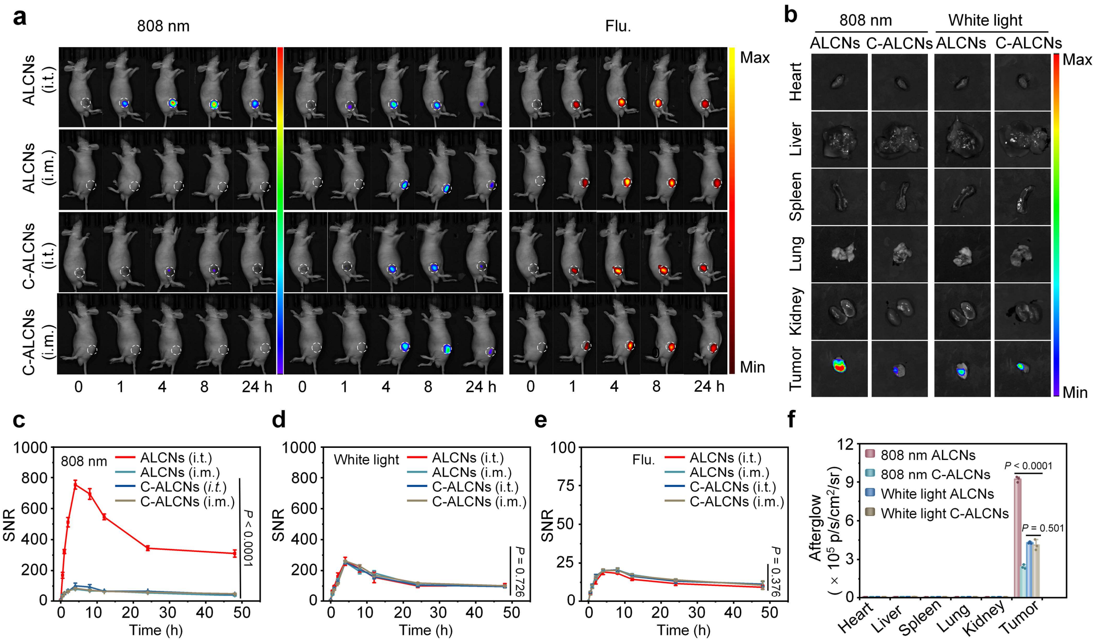

Supplementary Fig. 19. In vivo afterglow luminescence imaging of HepG2-xenograft tumors. a Afterglow images under an 808 nm pre-irradiation (left panel), white light pre-irradiation (middle panel), and fluorescence images (right panel) after intratumor and intramuscular injection of ALCNs or C-ALCNs (1 mg/kg ASN or C-ASN, and 0.1 mg/kg AIN). The fluorescence images were acquired with an excitation wavelength at 480 nm and emission wavelength at 580 nm. **b** Ex vivo afterglow images of various tissues under an 808 nm and white light pre-irradiation from HepG2-xenograft subcutaneous tumor-bearing mice at 48 h post-injection of ALCNs or C-ALCNs. **c-e** The quantified SNRs for afterglow luminescence imaging under an 808 nm pre-irradiation (c), white light pre-irradiation (d), fluorescence luminescence imaging (e) of HepG2-xenograft subcutaneous tumor-bearing mice as a function of time. **f** Quantification of ex vivo afterglow intensities of various tissues in Supplementary Fig. 19a. Data are presented as mean ± s.d. and analyzed by one-way ANOVA (n = 3 mice each group). Source data are provided as a Source Data file.

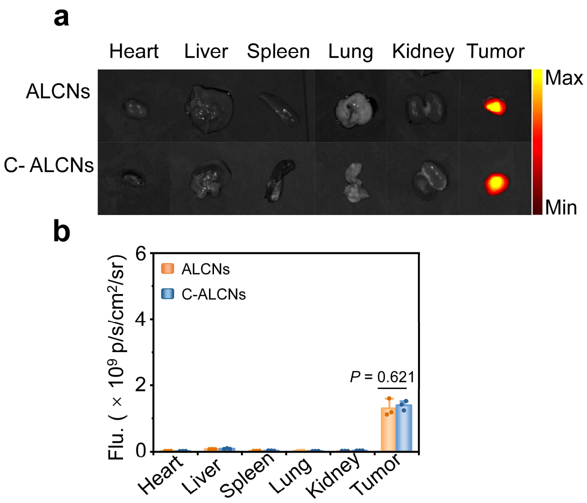

Supplementary Fig. 20. Study of biodistribution. a Ex vivo fluorescence images of various organs and tissues from an HepG2-xenograft subcutaneous tumor-bearing mouse intratumor injection of ALCNs and C-ALCNs (1 mg/kg ASN or C-ASN, and 0.1 mg/kg AIN) at 48 h with an excitation at 480 nm and emission at 580 nm. **b** Quantification of fluorescence intensities of organs and tissues. Data are presented as mean ± s.d. and analyzed by one-way ANOVA (n = 3 mice each group). Source data are provided as a Source Data file.

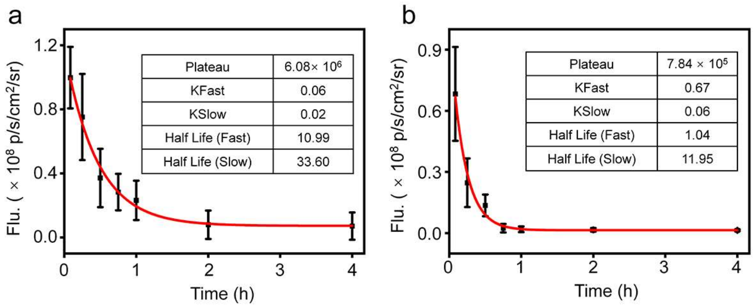

Supplementary Fig. 21. The time dependent blood clearance kinetics of AIN (0.8 mg/kg, with an excitation at 720 nm and emission at 790 nm) (a), and ASN (8 mg/kg, with an excitation at 480 nm and emission at 580 nm) (b). The data were fitted with a two-compartment model. Data are presented as mean ± s.d. (n = 3 mice each group). Source data are provided as a Source Data file.

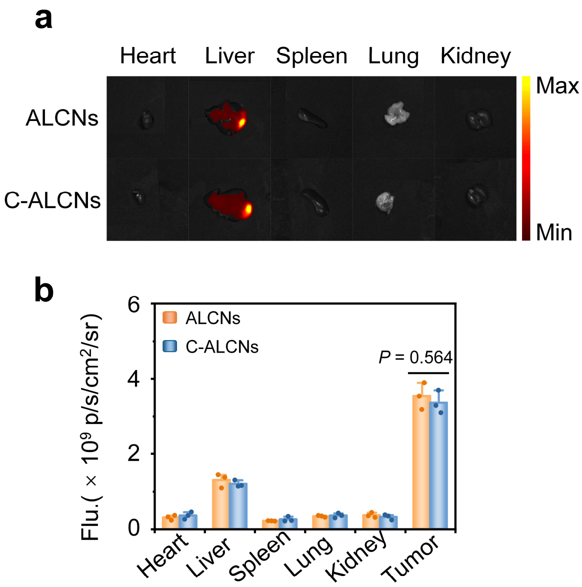

Supplementary Fig. 22. Study of biodistribution. a Ex vivo fluorescence images of various organs and tissues from an orthotopic liver tumor-bearing mouse intravenous injection of ALCNs and C-ALCNs (4 mg/kg ASN or C-ASN, and 0.4 mg/kg AIN) at 48 h with an excitation at 480 nm and emission at 580 nm. **b** Quantification of fluorescence intensities of organs and tissues. Data are presented as mean ± s.d. and analyzed by one-way ANOVA (n = 3 mice each group). Source data are provided as a Source Data file.

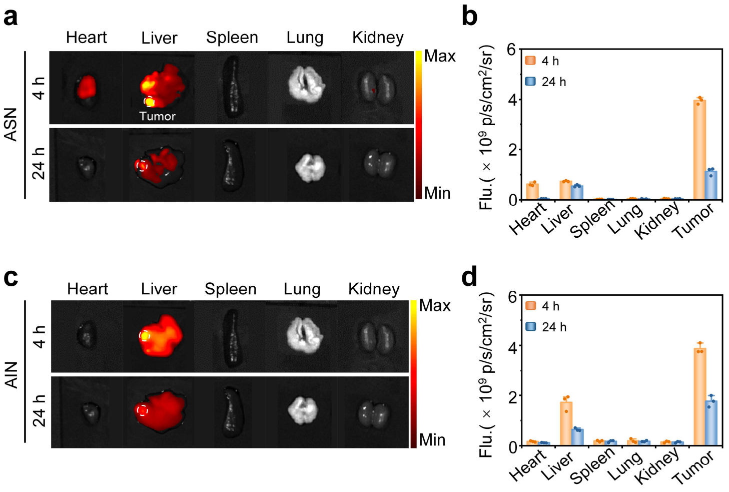

Supplementary Fig. 23. Biodistribution of ASN and AIN in various organs of orthotopic liver tumors. a, c Fluorescence images of ASN (8 mg/kg, with an excitation at 480 nm and emission at 580 nm) and AIN (0.8 mg/kg, with an excitation at 720 nm and emission at 790 nm) in heart, liver, spleen, lungs, kidney, and tumor at 4 h and 24 h. **b, d** Qualifications of fluorescence intensity in (a) and (c). Data are presented as mean ± s.d. (n = 3 mice each group). Source data are provided as a Source Data file.

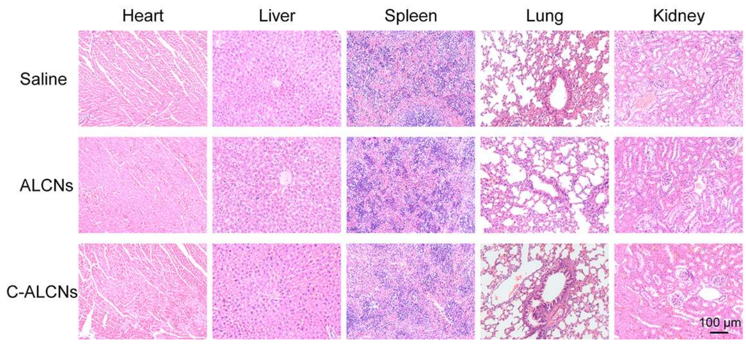

Supplementary Fig. 24. Study of biocompatibility. Representative H&E staining of the slices of mice organs harvested 1 d after systematic administration of ALCNs and C-ALCNs (200 μg/mL, 200 μL) with saline serving as control.

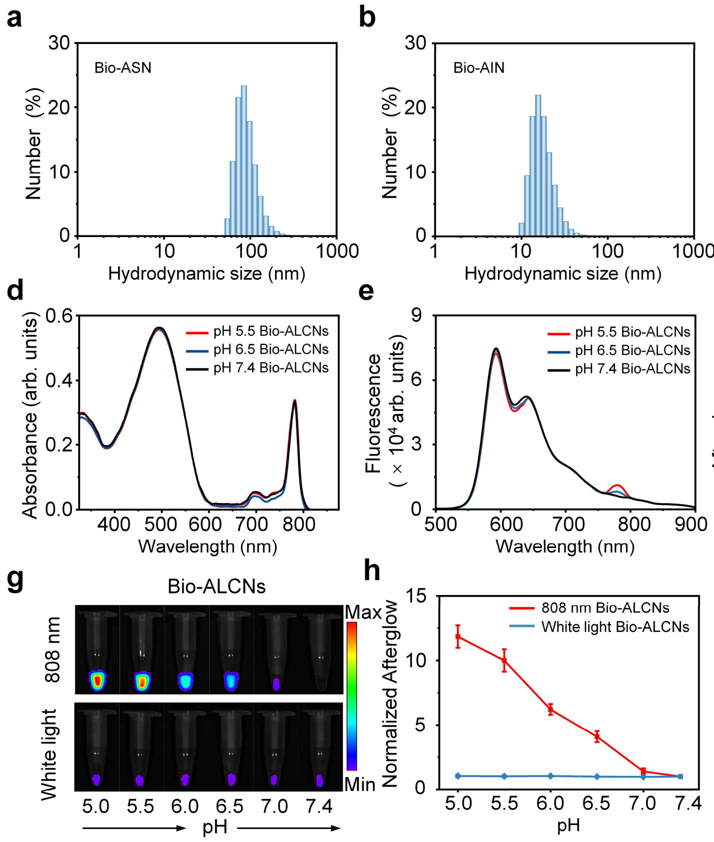

Supplementary Fig. 25. Characterization and study of the pH-responsive activatable upconversion afterglow luminescence of Bio-ALCNs. a, b Average hydrodynamic diameters of Bio-ASN (a), and Bio-AIN (b) in 1 × HEPES buffer (pH = 7.4). **c** Time-dependent zeta potential changes of Bio-ASN (10 μg/mL) at different pH values in 1 × HEPES buffer (*n* = 3 independent experiments). **d** UV-Vis absorption spectra of Bio-ALCNs (10 μg/mL Bio-ASN and 1 μg/mL Bio-AIN) at different pH values in 1 × HEPES buffer. **e** Fluorescence spectra of Bio-ALCNs (10 μg/mL Bio-ASN and 1 μg/mL Bio-AIN) at different pH values in 1 × HEPES buffer with an excitation wavelength at 480 nm. **f** Afterglow spectra of Bio-ALCNs (10 μg/mL Bio-ASN, and 1 μg/mL Bio-AIN) at different pH values in 1 × HEPES buffer. **g** Afterglow luminescence under 808 nm laser pre-irradiation (upper panel) and under white light pre-irradiation (lower panel) images of Bio-ALCNs (20 μg/mL Bio-ASN and 2 μg/mL Bio-AIN) at different pH values in 1× HEPES buffer **h** Normalized afterglow intensities of Bio-ALCNs (20 μg/mL Bio-ASN and 2 μg/mL Bio-AIN) under 808 nm laser and white light pre-irradiation (*n* = 3 independent experiments). **i** Fitted calibration curve for the afterglow intensities of Bio-

ALCNs as a function of pH values. Data are presented as mean ± s.d. (n = 3 independent experiments). Source data are provided as a Source Data file.

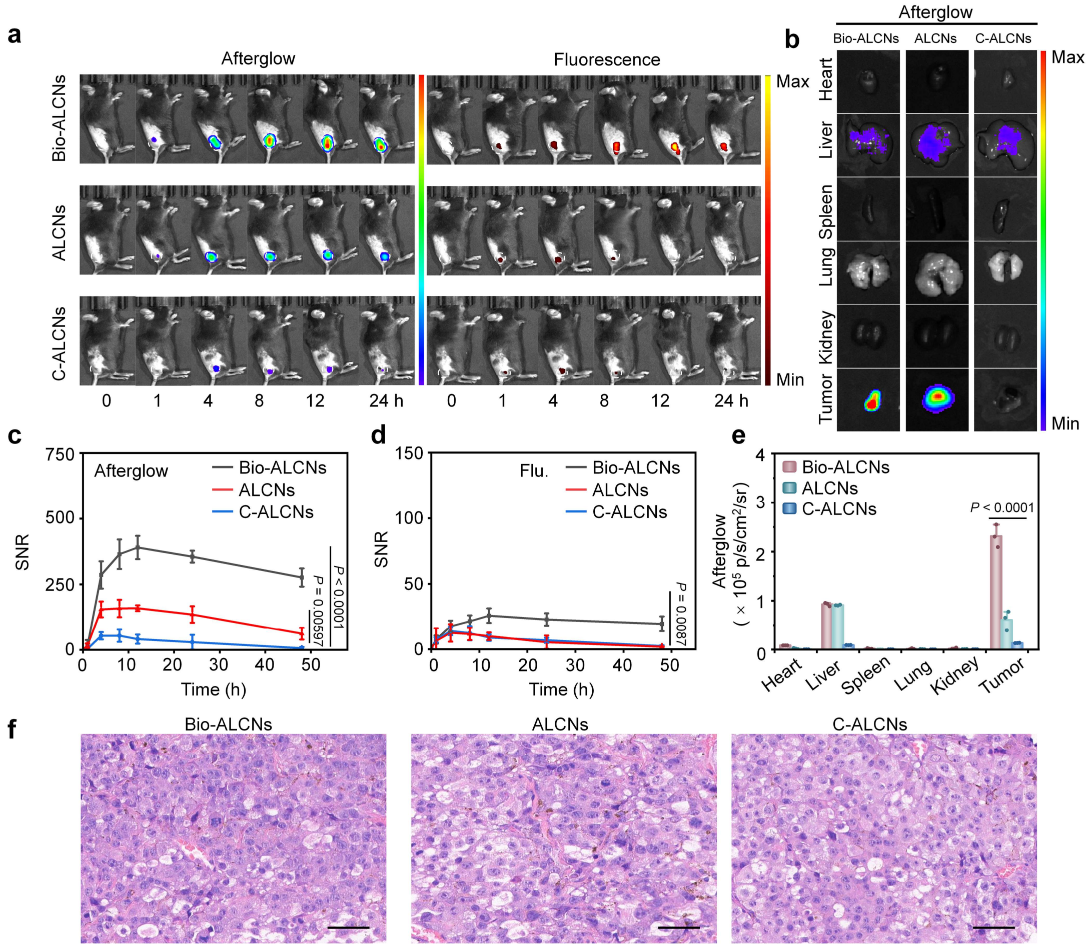

Supplementary Fig. 26. In vivo activatable afterglow imaging of B16F10-xenograft tumors. a Afterglow images under an 808 nm pre-irradiation (left panel), and fluorescence images (right panel) after intravenous injection of Bio-ALCNs, ALCNs or C-ALCNs (4 mg/kg Bio-ASN, ASN or C-ASN, and 0.4 mg/kg Bio-AIN, or AIN). The fluorescence images were acquired with an excitation wavelength at 480 nm and emission wavelength at 580 nm. **b** Ex vivo afterglow images of various tissues under an 808 nm laser pre-irradiation from B16F10 tumor-bearing mice at 48 h post-injection of Bio-ALCNs, ALCNs or C-ALCNs intravenously. **c, d** The quantified SNRs for afterglow luminescence imaging under 808 nm pre-irradiation (c), or fluorescence imaging (d) of B16F10-tumor-bearing mice as a function of time. **e** Quantification of ex vivo afterglow intensities of various tissues in **b**. **f** H&E staining images of tumor slices of mice after injection with Bio-ALCNs, ALCNs or C-ALCNs (N: normal tissue, T: tumor tissue). The scale bar represents 100 μm. Data are presented as mean ± s.d. and analyzed by one-way ANOVA (n = 3 mice each group). Source data are provided as a Source Data file.

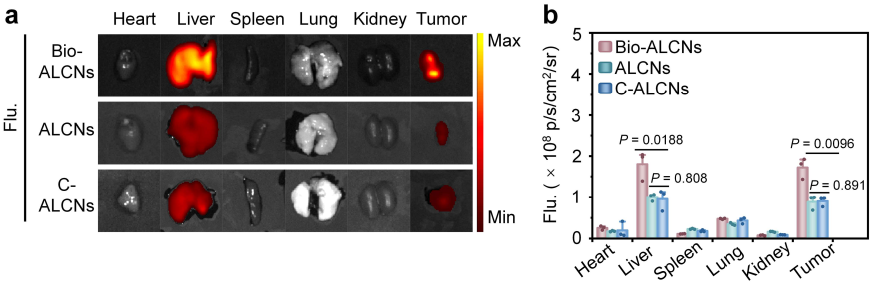

Supplementary Fig. 27. Study of biodistribution. a Ex vivo fluorescence images of various organs and tissues from B16F10 tumor-bearing mouse after intravenous injection of Bio-ALCNs, ALCNs, and C-ALCNs (4 mg/kg ASN or C-ASN, and 0.4 mg/kg AIN) at 48 h with an excitation at 480 nm and emission at 580 nm. **b** Quantification of fluorescence intensities of organs and tissues. Data are presented as mean ± s.d. and analyzed by one-way ANOVA (*n* = 3 mice each group). Source data are provided as a Source Data file.

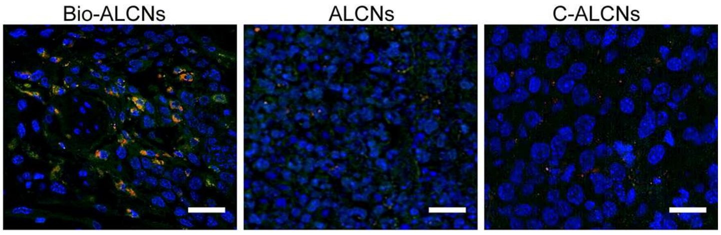

Supplementary Fig. 28. Fluorescence imaging of B16F10-xenograft tumor sections. Fluorescent images of B16F10 subcutaneous tumor sections after treatment with Bio-ALCNs, ALCNs or C-ALCNs scanning by Digital pathology scanner. ASN (green, 4 mg/kg, with an excitation at 490 nm and emission at 570 ± 10 nm), AIN (red, 0.4 mg/kg, with an excitation at 750 nm and emission at 780 ± 10 nm), nucleus (blue, 10 µM, with an excitation at 350 nm and emission at 420 ± 10 nm). The scale bar represents 10 µm.

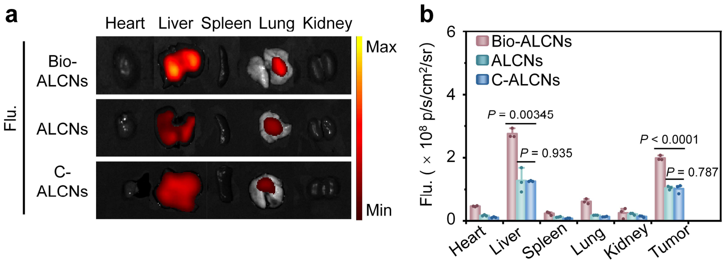

Supplementary Fig. 29. Study of biodistribution. a Ex vivo fluorescence images of various organs and tissues from pulmonary metastasis tumor-bearing mouse after intravenous injection of Bio-ALCNs, ALCNs and C-ALCNs (4 mg/kg Bio-ASN, ASN, or C-ASN, and 0.4 mg/kg AIN) at 48 h with an excitation at 480 nm and emission at 580 nm. **b** Quantification of fluorescence intensities of organs and tissues. Data are presented as mean ± s.d. and analyzed by one-way ANOVA (*n* = 3 mice each group). Source data are provided as a Source Data file.

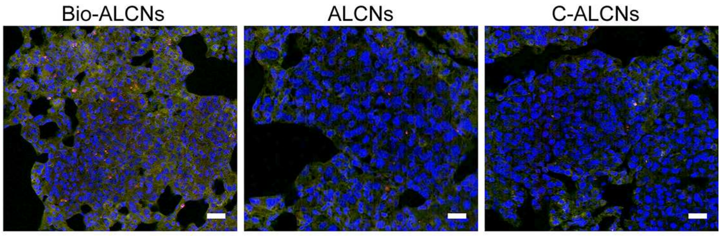

Supplementary Fig. 30. Fluorescence imaging of 4T1 pulmonary metastasis tumor sections. Fluorescent images of 4T1 pulmonary metastasis tumor sections after treatment with Bio-ALCNs, ALCNs or C-ALCNs scanning by Digital pathology scanner. Bio-ASN, ASN (green, 4 mg/kg, with an excitation at 490 nm and emission at 570 ± 10 nm), Bio-AIN, AIN (red, 0.4 mg/kg, with an excitation at 750 nm and emission at 780 ± 10 nm), nucleus (blue, 10 µM, with an excitation at 350 nm and emission at 420 ± 10 nm). The scale bar represents 10 µm.

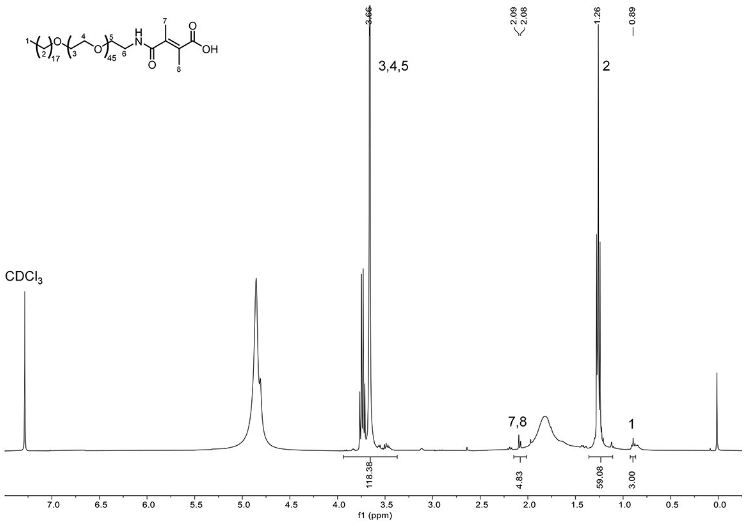

Supplementary Fig. 31. ^{1}H NMR spectrum of C_{18}-PEG_{2000}-DA in $CDCl_3$.

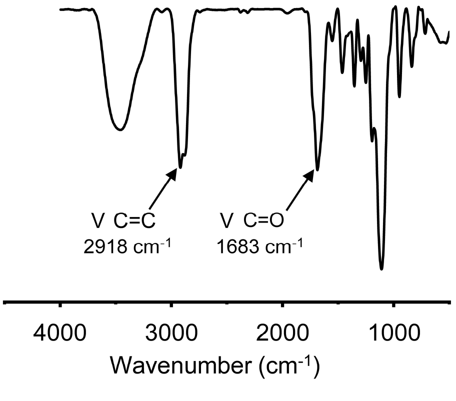

Supplementary Fig. 32. The FTIR spectrum of C_{18}-PEG_{2000}-DA mixed in a potassium bromide tablet.

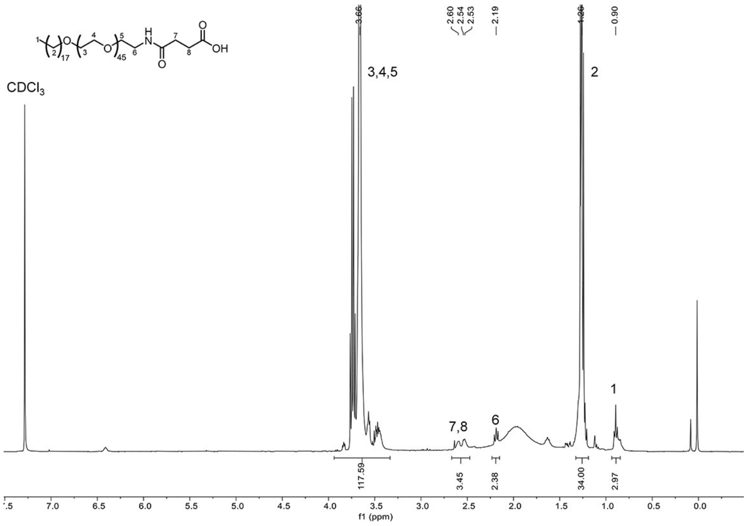

Supplementary Fig. 33. ^{1}H NMR spectrum of C_{18}-PEG_{2000}-SA in $CDCl_3$.

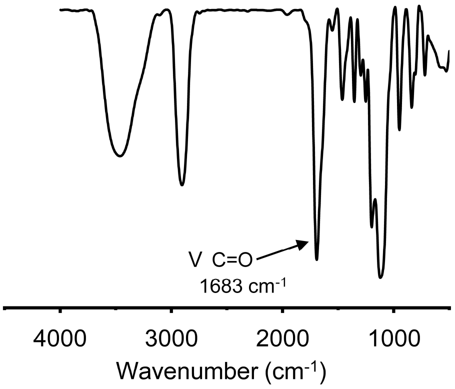

Supplementary Fig. 34. The FTIR spectrum of C_{18}-PEG_{2000}-SA mixed in a potassium bromide tablet.

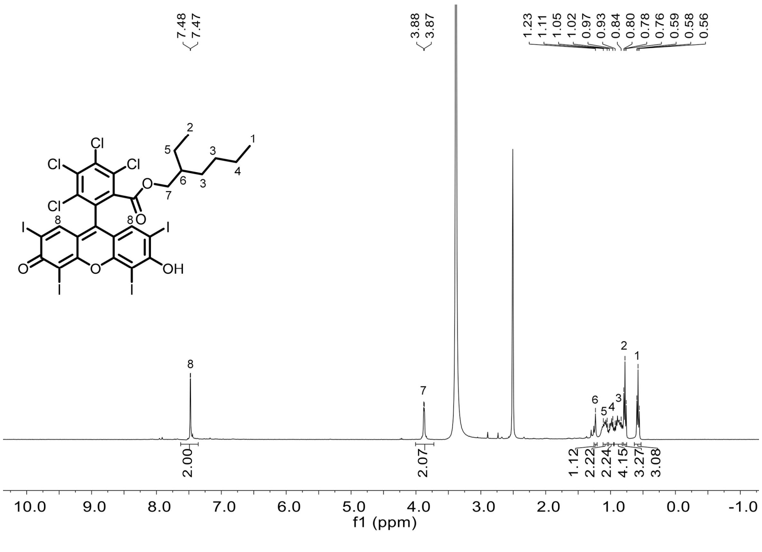

Supplementary Fig. 35. ^{1}H NMR spectrum of a-RB in DMSO-d_6.

Supplementary Table 1. SNR of representative molecular afterglow imaging probes.

System	λ_{em} (nm)	SNR in mice (administration/dose)	Ref.
**PPV and its analogs**			
MEHPPV/NCBS/PEG-*b*-PPG-*b*-PEG NPs	780	419 (s.c./12.5 μg)	1
PPV-TPP/PEG-*b*-PPG-*b*-PEG NPs	720	27.6 (i.v./80 μg)	2
PPV-PEGL/NCBS NPs	775	4170 (s.c./6.5 μg)	3
PFPV/TTMN/PEG-*b*-PPG-*b*-PEG NPs	630	—	4
**Thiophene-based SPs**			
PFODBT NPs	690-770	50 (i.t./60 μg）	5
NIR-3 NPs	800-820	150 (i.t./50 μg）	6
**AEE**			
AEE-1	< 400	150 (i.v./95.3 μg)	7
AEE-2/TPE-TV-CyP/DSPE-PEG NPs	625	461.3 (s.c./15 μg)	8
AEE-4/TPE-Ph-DCM/DSPE-PEG NPs	640	—	9
AEE-5/$(TPE\text{-}DPA)_2$-Py/DSPE-PEG NPs	660	—	10
AEE-6	600	93.7 (blood/20 μM)	11
AEE-7/MB	700	—	12
**DO and SO**			
DO/NCBS/PFVA/PEG-*b*-PPG-*b*-PEG NPs	780	2922 (i.v./95.3 μg)	13
SO/PdPc$(Obu)_8$/Eu$(TPPO)_2$(β-NTA)$_3$ NPs	613	—	14
CUEM/SiPc/carboxylated polystyrene NPs	445	131 (s.c./150 μg)	15
**CLA**			
ADLumin-1	660	2189 (s.c./-)	16
C_8-CLA/NCBS/TTB/DSPE-PEG NPs	780	18 (i.v./70 μg)	17
**Porphyrin**			
Ppa nanomicelles	760	215.1 (i.v./224 μg)	18
Ce4/PEG-*b*-PPG-*b*-PEG NPs	660	690 (s.c./1.25 μg)	19
**Rubrene**			
Rubrene/Ir-OTf/PEG-*b*-PPG-*b*-PEG NPs	560	4 (i.p./4.2 mg)	20
**This work**			
ALCNs	590	632.3 (i.t./16.8 μg)<br>224.3(i.v./67.2 ug)	

3. Supplementary References

1. Miao, Q. et al. Molecular afterglow imaging with bright, biodegradable polymer nanoparticles. *Nat. Biotechnol.* **35**, 1102-1110 (2017).

2. Cui, D., Xie, C., Li, J., Lyu, Y. & Pu, K. Semiconducting photosensitizer-incorporated copolymers as near-infrared afterglow nanoagents for tumor imaging. *Adv. Healthc. Mater.* **7**, 1800329 (2018).

3. Xie, C., Zhen, X., Miao, Q., Lyu, Y. & Pu, K. Self-assembled semiconducting polymer nanoparticles for ultrasensitive near-infrared afterglow imaging of metastatic tumors. *Adv. Mater.* **30**, 1801331 (2018).

4. Xu, Y. et al. An aggregation-induced emission dye-powered afterglow luminogen for tumor imaging. *Chem. Sci.* **11**, 419-428 (2020).

5. Wang, Y. et al. Cyclic amplification of the afterglow luminescent nanoreporter enables the prediction of anti-cancer efficiency. *Angew. Chem. Int. Ed.* **60**, 19779-19789 (2021).

6. Liao, S. et al. A novel afterglow nanoreporter for monitoring cancer therapy. *Theranostics* **12**, 6883-6897 (2022).

7. Jiang, Y. et al. A generic approach towards afterglow luminescent nanoparticles for ultrasensitive in vivo imaging. *Nat. Commun.* **10**, 2064 (2019).

8. Chen, C. et al. Amplification of activated near-Infrared afterglow luminescence by introducing twisted molecular geometry for understanding neutrophil-involved diseases. *J. Am. Chem. Soc.* **144**, 3429-3441 (2022).

9. Ni, X. et al. Near-infrared afterglow luminescent aggregation-induced emission dots with ultrahigh tumor-to-liver signal ratio for promoted image-guided cancer surgery. *Nano Lett.* **19**, 318-330 (2019).

10. Gao, Z. et al. An activatable near-infrared afterglow theranostic prodrug with self-sustainable magnification effect of immunogenic cell death. *Angew. Chem. Int. Ed.* **61**, e202209793 (2022).

11. Yuan, H. et al. Afterglow amplification for fast and sensitive detection of porphyria in whole blood. *ACS Appl. Mater. Interfaces* **13**, 27991-27998 (2021).

12. Yang, M., Zhang, J., Shabat, D., Fan, J. & Peng, X. Near-infrared chemiluminescent probe for real-time monitoring singlet oxygen in cells and mice model. *ACS Sens.* **5**, 3158-3164 (2020).

13. Zhang, F. et al. Afterglow implant for arterial embolization and intraoperative imaging. *Chem. Eur. J.* **28**, e202103795 (2022).

14. Wang, X., Yuan, W., Xu, M., Su, X. & Li, F. Visualization of acute inflammation through a macrophage-camouflaged afterglow nanocomplex. *ACS Appl. Mater. Interfaces* **14**, 259-267 (2022).

15. Su, X. et al. Enhanced blue afterglow through molecular fusion for bio-applications. *Angew. Chem. Int. Ed.* **61**, e202201630 (2022).

16. Zheng, X. et al. Organic nanoparticles with persistent luminescence for in vivo afterglow imaging-guided photodynamic therapy. *Chem. Eur. J.* **27**, 6911-6916 (2021).

17. Chen, W. et al. Near-infrared afterglow luminescence of chlorin nanoparticles for ultrasensitive in vivo imaging. *J. Am. Chem. Soc.* **144**, 6719-6726 (2022).

18. Duan, X. et al. Activatable persistent luminescence from porphyrin derivatives and supramolecular probes with imaging-modality transformable characteristics for improved biological applications. *Angew. Chem. Int. Ed.* **61**, e202116174 (2022).

19. Anjong, T. et al. Multifunction-harnessed afterglow nanosensor for molecular imaging of acute kidney injury in vivo. *Small* **18**, 2200245 (2022).

20. Liu, Y., Teng, L., Lou, X., Zhang, X. & Song, G. "Four-In-One" design of a hemicyanine-based modular scaffold for high-contrast activatable molecular afterglow imaging. *J. Am. Chem. Soc.* **145**, 5134-5144 (2023).
