# Supplementary material for: Acidity-activatable upconversion afterglow luminescence cocktail nanoparticles for ultrasensitive in vivo imaging
Source: Nat Commun. 2024 Mar 8;15:2124. doi: 10.1038/s41467-024-46436-z (PMC10923940; doi:10.1038/s41467-024-46436-z)
Supplement: Supplementary file 2 — Reporting Summary [file 41467_2024_46436_MOESM2_ESM.pdf]

nature portfolio

Corresponding author(s): Qingqing Miao

Last updated by author(s): Feb 18, 2024

Reporting Summary

Nature Portfolio wishes to improve the reproducibility of the work that we publish. This form provides structure for consistency and transparency in reporting. For further information on Nature Portfolio policies, see our Editorial Policies and the Editorial Policy Checklist.

Statistics

For all statistical analyses, confirm that the following items are present in the figure legend, table legend, main text, or Methods section.

n/a	Confirmed	
☐	☒	The exact sample size (*n*) for each experimental group/condition, given as a discrete number and unit of measurement
☐	☒	A statement on whether measurements were taken from distinct samples or whether the same sample was measured repeatedly
☐	☒	The statistical test(s) used AND whether they are one- or two-sided<br>*Only common tests should be described solely by name; describe more complex techniques in the Methods section.*
☐	☒	A description of all covariates tested
☐	☒	A description of any assumptions or corrections, such as tests of normality and adjustment for multiple comparisons
☐	☒	A full description of the statistical parameters including central tendency (e.g. means) or other basic estimates (e.g. regression coefficient) AND variation (e.g. standard deviation) or associated estimates of uncertainty (e.g. confidence intervals)
☐	☒	For null hypothesis testing, the test statistic (e.g. *F*, *t*, *r*) with confidence intervals, effect sizes, degrees of freedom and *P* value noted<br>*Give P values as exact values whenever suitable.*
☒	☐	For Bayesian analysis, information on the choice of priors and Markov chain Monte Carlo settings
☒	☐	For hierarchical and complex designs, identification of the appropriate level for tests and full reporting of outcomes
☐	☒	Estimates of effect sizes (e.g. Cohen's *d*, Pearson's *r*), indicating how they were calculated

Our web collection on statistics for biologists contains articles on many of the points above.

Software and code

Policy information about availability of computer code

Data collection	Living Image Software (Perkin Elmer) was used for IVIS Imaging System to acquire afterglow and fluorescence imaging data.
Data analysis	All software used for date analysis are fully described in the materials and methods of manuscript. Afterglow and fluorescence imaging data were analyzed using Living Image 4.5 Software (Perkin Elmer). Confocal fluorescence microscopy imaging data were analyzed using confocal laser scanning microscope (OLYMPUS, FV1200). Fluorescence images of tumor slices were analyzed by Digital Pathology Scanner (FK-FL-020). All statistical calculations were performed using Origin 2024 (64-bit).

For manuscripts utilizing custom algorithms or software that are central to the research but not yet described in published literature, software must be made available to editors and reviewers. We strongly encourage code deposition in a community repository (e.g. GitHub). See the Nature Portfolio guidelines for submitting code & software for further information.

Data

Policy information about availability of data

All manuscripts must include a data availability statement. This statement should provide the following information, where applicable:

- Accession codes, unique identifiers, or web links for publicly available datasets
- A description of any restrictions on data availability
- For clinical datasets or third party data, please ensure that the statement adheres to our policy

The data supporting the findings of this study are available within the paper and Supplementary Information files. Any raw data files in another format are available from the corresponding author. Source data are provided with this paper.

Research involving human participants, their data, or biological material

Policy information about studies with human participants or human data. See also policy information about sex, gender (identity/presentation), and sexual orientation and race, ethnicity and racism.

Reporting on sex and gender	N/A
Reporting on race, ethnicity, or other socially relevant groupings	N/A
Population characteristics	N/A
Recruitment	N/A
Ethics oversight	N/A

Note that full information on the approval of the study protocol must also be provided in the manuscript.

Field-specific reporting

Please select the one below that is the best fit for your research. If you are not sure, read the appropriate sections before making your selection.

☒ Life sciences ☐ Behavioural & social sciences ☐ Ecological, evolutionary & environmental sciences

For a reference copy of the document with all sections, see nature.com/documents/nr-reporting-summary-flat.pdf

Life sciences study design

All studies must disclose on these points even when the disclosure is negative.

Sample size	We used G*power analysis to ensure that sample sizes were sufficient for the statistics to have adequate power (over 80%).
Data exclusions	No data were excluded from the study.
Replication	Experiments were repeated at least three independent experiments with similar results. All experiments were reproduced to reliably support conclusions stated in the manuscript.
Randomization	The cages of mice were randomly selected and then divided into experimental groups for further treatment.
Blinding	The investigators were blinded to group allocation during the experiments.

Reporting for specific materials, systems and methods

We require information from authors about some types of materials, experimental systems and methods used in many studies. Here, indicate whether each material, system or method listed is relevant to your study. If you are not sure if a list item applies to your research, read the appropriate section before selecting a response.

Materials & experimental systems

n/a	Involved in the study
☒	☐ Antibodies
☐	☒ Eukaryotic cell lines
☒	☐ Palaeontology and archaeology
☐	☒ Animals and other organisms
☒	☐ Clinical data
☒	☐ Dual use research of concern
☒	☐ Plants

Methods

n/a	Involved in the study
☒	☐ ChIP-seq
☒	☐ Flow cytometry
☒	☐ MRI-based neuroimaging

Eukaryotic cell lines

Policy information about cell lines and Sex and Gender in Research

Cell line source(s)	4T1, 4T1-Luc mouse breast cancer cells, B16F10 mouse melanoma cells, and HepG2 human liver cancer cells were purchased from Stem Cell Bank, Chinese Academy of Sciences (Shanghai, China).
Authentication	All cell lines were authenticated by the supplier using Short Tandem Repeat test.
Mycoplasma contamination	No contamination was detected by the supplier (according to Hoechst DNA stains, agar culture and PCR-based assays).
Commonly misidentified lines (See ICLAC register)	No commonly misidentified cell lines were used.

Animals and other research organisms

Policy information about studies involving animals; ARRIVE guidelines recommended for reporting animal research, and Sex and Gender in Research

Laboratory animals	BALB/c-Nu male mice, BALB/c female mice, and C57B/J female mice (8-week-old) were purchased from Changzhou Cavens Laboratory Animal Co., Ltd. (Jiangsu, China) and used for all the in vivo experiments. And all mice were housed under standard conditions (25 ± 3 °C, 60 ± 10% relative humidity) with 12 h light/dark cycle.
Wild animals	The study did not involve wild animals.
Reporting on sex	In 4T1 subcutaneous tumor, pulmonary metastasis tumor, HepG2 subcutaneous tumor and B16F10 subcutaneous tumor models, female mice were involved. Male mice were used for establishing HepG2 orthotopic tumor models, since male mice were better able to withstand abdominal surgery. This study results were independent of sex or gender.
Field-collected samples	The study did not involve samples collected from the field.
Ethics oversight	All animal experiments conducted in this study were carried out following the guidelines established by the Care and Use of Laboratory Animals of Soochow University and were approved by the Animal Ethics Committee of the Soochow University Laboratory Animal Center (Suzhou, China, 202110A0067).

Note that full information on the approval of the study protocol must also be provided in the manuscript.

Plants

Seed stocks	N/A
Novel plant genotypes	N/A
Authentication	N/A
